# Evaluation of a novel technology-supported fall prevention intervention – study protocol of a multi-centre randomised controlled trial in older adults at increased risk of falls

**DOI:** 10.1186/s12877-023-03810-8

**Published:** 2023-02-18

**Authors:** Tobias Morat, Melina Snyders, Philipp Kroeber, Alice De Luca, Valentina Squeri, Martin Hochheim, Philipp Ramm, Annika Breitkopf, Michael Hollmann, Wiebren Zijlstra

**Affiliations:** 1 Institute of Movement and Sport Gerontology, German Sport University (GSU) Cologne, Am Sportpark Muengersdorf 6, 50933 Cologne, Germany; 2 Movendo Technology (MT), Calata Cattaneo 15, 16128 Genoa, Italy; 3 Generali Health Solutions GmbH (GHS), Hansaring 40-50, 50670 Cologne, Germany; 4 FPZ GmbH, Gustav-Heinemann-Ufer 88a, 50968 Cologne, Germany

**Keywords:** Fall prevention, Technology-based, Randomised controlled trial, RCT, Robot device

## Abstract

**Background:**

Increasing number of falls and fall-related injuries in an aging society give rise to the need for effective fall prevention and rehabilitation strategies. Besides traditional exercise approaches, new technologies show promising options for fall prevention in older adults. As a new technology-based approach, the hunova robot can support fall prevention in older adults.

The objective of this study is to implement and evaluate a novel technology-supported fall prevention intervention using the hunova robot compared to an inactive control group. The presented protocol aims at introducing a two-armed, multi-centre (four sites) randomised controlled trial, evaluating the effects of this new approach on the number of falls and number of fallers as primary outcomes.

**Methods:**

The full clinical trial incorporates community-dwelling older adults at risk of falls with a minimum age of 65 years. Including a one-year follow-up measurement, all participants are tested four times. The training programme for the intervention group comprises 24-32 weeks in which training sessions are scheduled mostly twice a week; the first 24 training sessions use the hunova robot, these are followed by a home-based programme of 24 training sessions. Fall-related risk factors as secondary endpoints are measured using the hunova robot. For this purpose, the hunova robot measures the participants’ performance in several dimensions. The test outcomes are input for the calculation of an overall score which indicates the fall risk. The hunova-based measurements are accompanied by the timed-up-and-go test as a standard test within fall prevention studies.

**Discussion:**

This study is expected to lead to new insights which may help establish a new approach to fall prevention training for older adults at risk of falls. First positive results on risk factors can be expected after the first 24 training sessions using the hunova robot. As primary outcomes, the number of falls and fallers within the study (including the one-year follow-up period) are the most relevant parameters that should be positively influenced by our new approach to fall prevention. After the study completion, approaches to examine the cost-effectiveness and develop an implementation plan are relevant aspects for further steps.

**Trial registration:**

German Clinical Trial Register (DRKS), ID: DRKS00025897. Prospectively registered 16 August 2021, https://drks.de/search/de/trial/DRKS00025897.

**Supplementary Information:**

The online version contains supplementary material available at 10.1186/s12877-023-03810-8.

## Background

Age-associated changes in functioning lead to a higher incidence of falls in older adults. Studies indicate that approximately one in three adults (i.e., more than 30%) aged at least 65 falls once per year [1–3]. With increasing age after 65, or in the presence of specific age-related diseases, fall risk may even increase beyond 50% [1–3]. The individual consequences of falls may comprise fall-related injuries, subsequent activity restriction, and fear of falling [4–6]. In addition to individual costs, the societal costs of falls are enormous; for Germany, direct health care costs after a fall-related fracture of the femoral neck have been estimated at approximately 15 k euros per person [7]. According to estimates, the costs of direct medical treatment of hip fractures in Germany are more than one billion euros per year, not including long-term nursing costs and indirect costs such as time lost from work by relatives [8]. Thus, it is paramount to prevent older persons from falling. Fall risk is associated with measures from multiple domains; these include not only measures of cognitive and physical functioning but also gender, age, presence of co-morbidities, medication use, fear of falling, and environmental factors [9]. Not all variables associated with the incidence of falls can be influenced (e.g., gender, age). However, some of the most important predictors of fall risk, such as muscle function, balance control, and gait quality, can be improved by appropriate fall prevention interventions [10, 11].

Reviews of available studies demonstrate that effective fall prevention strategies simultaneously address different risk factors [10, 12, 13]. Exercise-based strategies are most effective when they combine balance and functional exercises plus resistance exercises [10]. Particularly, the integration of activities that are important for daily life functioning, such as rising from a chair are essential. According to current evidence, an exercise programme needs to have a minimal duration and has to be challenging in intensity to be effective in reducing the fall incidence [10]. Among the current challenges to prevent falls in older persons are a timely identification of persons at risk, as well as ensuring older persons’ participation and adherence to appropriate fall prevention programmes. To realise optimal effects, the type of exercises and their dosing should preferably be individualised, and the person should participate over a sufficiently long duration [14–16]. However, there is a large gap between evidence about effective fall prevention interventions and their transfer into clinical practice and preventing initial falls [17, 18]. In their review, Michael et al. [19] analysed the barriers most often noted by providers of primary care-relevant interventions to prevent falls. The participants (physicians) suggested that they would be more convinced to act in regard to fall prevention for older adults if they have actual data about falls of their patients and an effective fall-risk assessment and treatment. Furthermore, they would prefer information where they can refer high-risk patients to receive effective fall prevention [19]. With our study protocol we aim to address these omissions.

Miscellaneous new technology can contribute to the assessment of fall risk [20–22] as well as the implementation of novel fall prevention interventions [23–28]. Furthermore, stepping interventions [29] and perturbation-based interventions [30] are implemented to prevent falls. The effects of robot-assisted approaches using exoskeletons and (end-effector) robots on fall-related risk-factors have already been studied in different disease groups (multiple sclerosis, stroke, Parkinson’s disease) (e.g., [31–33]). However, there still is little information about the use of robotic devices to prevent falls in older adults. Only one study by Verrusio et al. [34] used robotic technology for fall prevention training with healthy older adults. Their results showed first positive effects of using the exoskeleton human body posturizer regarding fall incidence in older adults without any diseases [34].

The present study protocol aims to evaluate the contribution of such new technology to the prevention of falls. Recent developments have led to technology that can be used to assess individual fall risk profiles and/or implement exercise programmes which aim to address individual risk profiles [22, 26]. One specific example of such systems is the hunova robot (Movendo Technology, Genoa, Italy), a robotic medical device for the evaluation of sensory-motor functions and rehabilitation after injuries of ankle, lower leg, or trunk. By measuring various aspects of balance and lower limb functioning, the hunova device creates an individual fall risk profile and identifies deficient dimensions on which an individualisation of the training programme takes place. During the training sessions, participants receive live feedback on a screen, which can be implemented directly. All details can be found in Cella and colleagues and Saglia et al. [35, 36]. First clinical studies have demonstrated positive results in the rehabilitation of stroke patients, as well as in patients with Parkinson’s disease (PD) [37, 38]. As a new approach, we use a novel technology (hunova robot), developed and used previously for specific diseases, now moving into older adult research, particularly fall prevention.

This paper describes a study protocol that aims to evaluate the effects of a complex fall prevention intervention on fall incidence and fall risk in older persons with an increased fall risk. The intervention comprises two consecutive components; a technology-supported exercise programme followed by a home-based exercise programme. Based on a randomised controlled design, the study will evaluate the hypotheses that the intervention reduces fall incidence (primary outcome) and fall risk (secondary outcome).

## Methods/design

This full clinical trial complies with the revised Helsinki declaration (cf. Fortaleza, Brazil, 2013), as well as Germany’s laws for data security (i.e., General Data Protection Regulation of the German Data Protection Act). Ethical approval of the study has been obtained from the ethics committee of the German Sport University Cologne (GSU, application number 104/2021). This approval covers all four study sites (Cologne, Bonn, Dusseldorf, and Bremen; addresses are included at the end of the protocol).

The study was prospectively registered in the German Clinical Trial Register (registration number: DRKS00025897).

### Study design

**Fig. 1:**
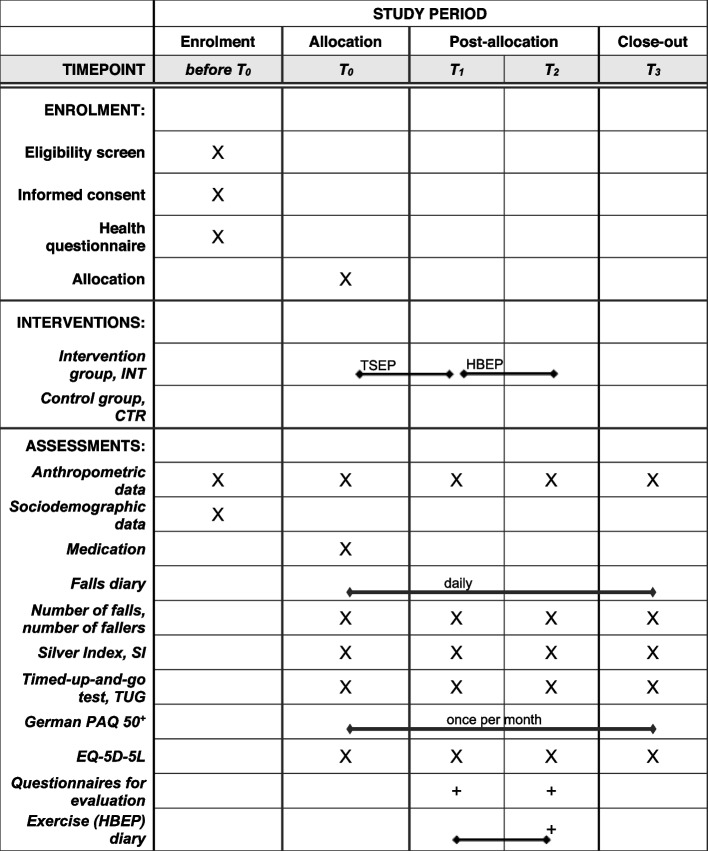
SPIRIT-Figure: Schedule of enrolment, interventions, and assessments (CTR = control group; HBEP = home-based exercise programme; INT = intervention group; TSEP = technology-supported exercise programme; X = relevant for INT and CTR; +  = only relevant for INT)

The study design is a multi-site randomised controlled longitudinal intervention study which includes four comprehensive assessments: T_0_, T_1_, T_2_, and T_3_ for all participants. All participants are randomly assigned to either the intervention group (INT) or the control group (CTR; see randomisation procedures). Figure 1 shows the SPIRIT-figure, including the schedule of enrolment, interventions, and assessments.

**Fig. 2:**
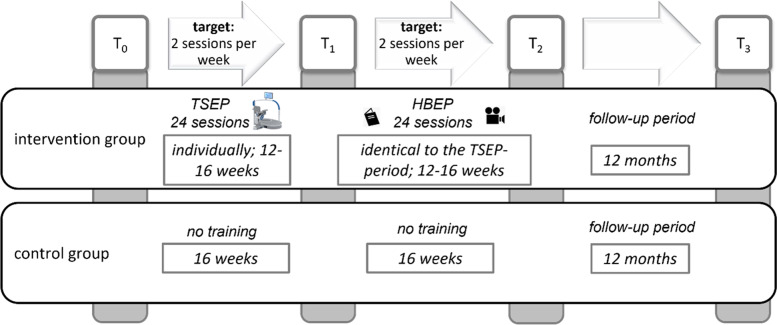
Timeline: T_0_ is a baseline assessment; T_1_ is an assessment after finishing the first technology-supported part of the intervention; T_2_ is an assessment after finishing the home-based exercise programme; and T_3_ is a final follow-up assessment 12 months after T_2_. HBEP = home-based exercise programme; TSEP = technology-supported exercise programme

For the intervention group, T_0_ is a baseline assessment; T_1_ an assessment after finishing the technology-supported part of the intervention; T_2_ an assessment after finishing the home-based exercise programme; and T_3_ a final follow-up assessment 12 months after T_2_. In total, the training period of the intervention group comprises 2*24 units (48 sessions in total) with a training frequency of two planned training sessions of 30 min each per week. The intervention period of the technology-supported exercise programme is individually dependent on the time progress of the completed 24 sessions. Given the defined training frequency of two training sessions per week, the technology-supported exercise programme has a minimum intervention period of 12 weeks. If individual training sessions are cancelled, the intervention period may be extended, however, it is limited to a maximum of 16 weeks. Identical to the intervention period of the technology-supported exercise programme, the period of the home-based programme is then determined individually for each participant. Participants in the control group receive all four measurements. Participants in the control group are not offered any actions; they are instructed to maintain their activities as usual. The time periods between the T_0_, T_1_ and T_2_ measurements are defined as 16 weeks for the control group. Both groups end at T_3_ with a final follow-up assessment 12 months after T_2_. Figure 2 shows the detailed timeline for study participants. Participants in the control group will have the opportunity to participate in an optimized training programme using the hunova robot after the end of the study.

### Study centres

All assessments, the technology-supported exercise programme (TSEP), as well as two sessions in which participants receive instructions for the home-based exercise programme (HBEP) take place in participating study centres in Cologne, Bonn, Dusseldorf, and Bremen, respectively (addresses are included at the end of this protocol). From each centre, 2-4 sport or physio-therapists take part in the research study. Before the start of the intervention, all participating therapists are trained. This training includes several e-learning (online) modules that must be completed before on-site training begins. All training content of this examination is developed by the German Sport University in cooperation with Movendo Technology. The on-site sessions will be implemented by a researcher from the German Sport University together with a Movendo Technology specialist before the study starts to have a high standardisation and fulfil quality criteria for each training and exercising person.

### Participants and recruitment

The target group for the intervention consists of community dwelling older persons aged at least 65 years at an increased risk of falls. Recruitment of potential participants will be through Generali Health Solutions (GHS), who will address insured persons of “Generali Deutschland Krankenversicherung AG” (Generali Germany Health Insurance AG) and its subsidiary. Insured persons receive a personalised invitation letter in which they are asked to participate in the study. If they do not contact any of the study partners within the first two weeks after the initial invitation letter, they will receive a reminder letter. If an insufficient number of appointments at T_0_ is made, invited insured persons will receive phone calls. Inclusion criteria: aged at least 65 years; an increased fall risk (defined via a composite risk score (see further text)), ability to walk more than 6 m without assistance, residence within a maximal distance of 20 kms (as the crow flies) to the closest study centre. Exclusion criteria are: a) body height < 1.50 m; body weight > 150 kg (the hunova robot was designed and sized taking into consideration these anthropometric measures for the target population of the device. These choices impacted both the mechanical design and the sizing design of the device); level of care in Germany between 2 and 5 (In Germany, a distinction is made between five levels of care. For classification into a care level, the independence and abilities in six areas of life (mobility, mental and communicative abilities, behaviour and psychological problems, self-care, independent handling of illness- or therapy-related demands and burdens, as well as coping with them, organisation of everyday life and social contacts) are assessed using an instrument based on nursing expertise. Points are awarded for these six areas, which are weighted differently and lead to an overall score (up to a maximum of 100). This score is used for classification into one of the five care levels, which range from care level 1 (minor impairments of independence or abilities) to care level 5 (most severe impairments that are accompanied by special requirements for nursing care). The care level assigned determines the care insurance benefits that the person in need of care receives [39]); incapacity to be physically active over at least 30 min; medical surgery within the last 12 months at the spine, hip, legs, knees, feet (e.g. medical surgery after femoral neck fracture, medical surgery for the purpose of joint replacement or joint stiffening); acute cancer disease including chemotherapy, radiotherapy, biological or palliative therapy; dementia; Parkinson’s disease; multiple sclerosis; epilepsy; cardiovascular disease (e.g. cardiac arrhythmias, heart failure, poorly controlled hypertension, chest pain at rest or on exertion); b) depression, anxiety disorder, schizophrenia, that is not treated properly; consequences of a stroke that impair motor and cognitive functions and/or limitations within speaking substantially; serious respiratory disease (e.g. COPD - chronic obstructive pulmonary disease); Menière’s disease or another disease associated with vertigo; injury or condition that limits the sensitivity and/or motor function of the legs or feet (e.g. spinal stenosis, back pain that radiates to the legs, acute disc prolapse); problems reading information on a screen about 1 m away (e.g. on TV or on a computer screen) -with or without glasses; acute inflammation of the musculoskeletal system (e.g. arthritis, rheumatism in acute attack, ankylosing spondylitis (Bechterew’s disease) in acute attack, acute attack of gout); deep vein thrombosis or "open" leg (e.g. ulcer); the need to wear custom-made orthopaedic footwear (this does not mean shoe inserts) due to sensitivity disorders in the feet; bone fracture due to osteoporosis or noticeable physical changes (sharp decrease in height, increasing development of a hunchback) (list from the health questionnaire). Diseases from a) are “hard” exclusion criteria. If one (or more) of the other b) diseases is present, interested persons are advised to call the Generali health phone hotline to check if a study participation is still possible. Together with the invitation letter, all identified insured persons receive all study materials (detailed study information brochure, health questionnaire, informed consent) by mail. Upon signing the informed consent, they can participate in the study. They will then be tested and, if eligibility is confirmed, then randomly assigned to either INT or CTR. Randomisation procedures, contents of the intervention, as well as the assessments for determining eligibility and evaluating the intervention are described in next subsections.

### Randomisation procedures

An equal number of participants in the intervention and control group will be determined for each study centre. For this purpose, all eligible participants draw a lot with either INT or CTR after the baseline assessment. The number of lots for INT and CTR is equal and limited. Drawn lots are not returned to the lottery pot until all lots have been drawn. The examiners of the centres have no influence on the allocation to the groups, a change between groups is not possible.

### Assessments

As part of screening for eligibility, date of birth, gender, body height and weight, data from a health questionnaire, as well as a fall risk profile and current medications, will be collected. The health questionnaire aims to identify the presence of medical conditions (e.g., cardiovascular or neurological disease, high blood pressure, osteoporosis, acute inflammatory conditions, herniated disk, hip fractures, vision deficits). The fall risk profile is based on a composite risk score defined as the “Silver Index” (SI) which is calculated using specific parameters of the hunova robot [35]. The model is presented and validated by Cella et al. [35].

To evaluate intervention effects, a comprehensive assessment strategy which includes the SI, and a mobility test will be repeated at T_0_, T_1_, T_2_, and T_3_ (see Fig. 1 and 2). In addition to these repeated assessments, participants in the study will be instructed to keep a fall diary throughout the complete study (i.e., between T_0_ and T_3_).

#### Silver Index (SI)

Inputs into the SI are age, number of falls in the last 12 months, and the outcomes of following 8 tests for gait, balance, and rising from a chair (for detailed test procedures, and the calculation of the SI, see [35]:

Gait:

- 1. a 6-m walk test is used for determining gait speed;

Balance control is tested while the participant is standing on a force platform (foot platform) under the following conditions:

- 2. quiet standing with eyes open on static foot platform (30 s);
- 3. quiet standing with eyes closed on static foot platform (30 s);
- 4. standing with eyes open on an unstable foot platform (responding to the participant’s postural oscillations; 30 s);
- 5. standing with eyes open while the foot platform is moving along a default trajectory (30 s);
- 6. reactive balance: standing with eyes open while the foot platform implements specific perturbations (with impulses of 6 degrees in random directions and in random order;
- 7. limits of stability test: the person is instructed to move his/her body’s centre of mass as far as possible in anterior–posterior and medio-lateral directions without needing to step to maintain balance;

Muscle function (lower extremities):

- 8. the Five Times Sit to Stand Test (FTSST) is used as a functional test for lower extremity muscle function.

**Fig. 3:**
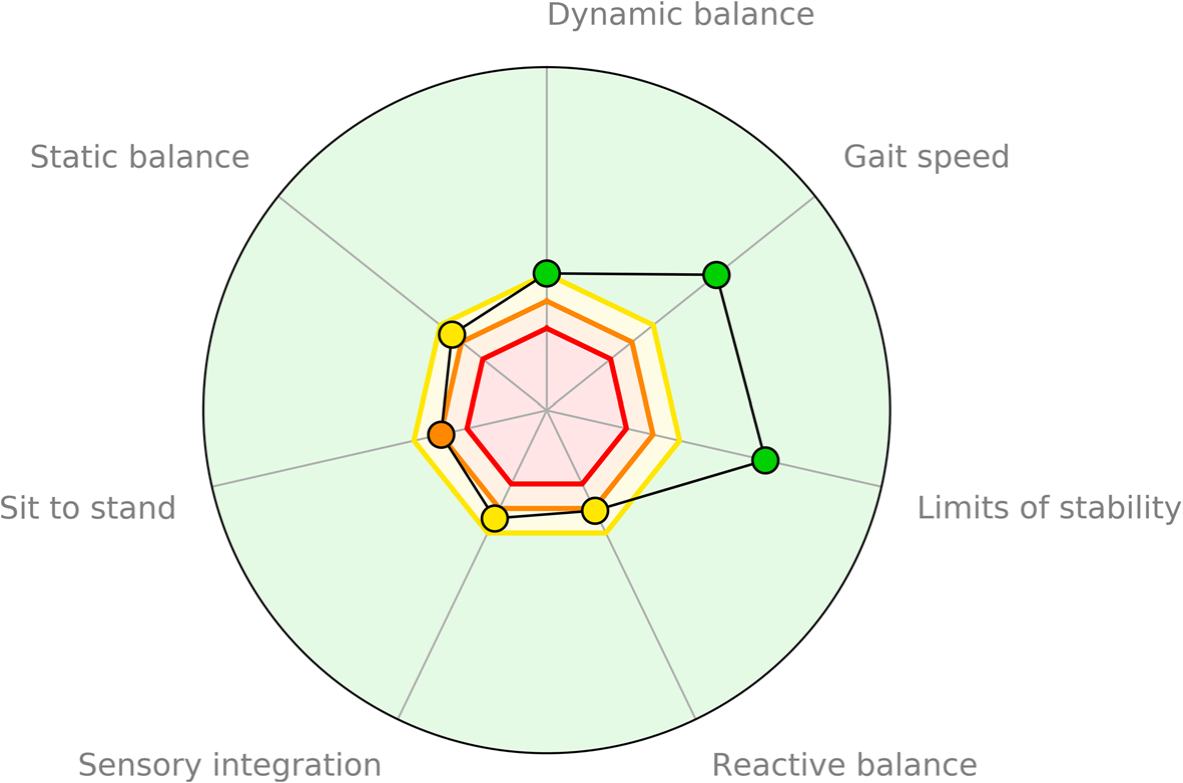
Radar plot as a result of the SI (Silver Index) assessment. The seven SI dimensions are displayed regarding the performance of the participants (green = normal (on average) meaning no deficit; yellow = poor performance; orange = very poor performance; red deficit = very very poor performance)

Based on these tests, the SI indicating fall risk was calculated [35]. SI ranges between 0 and 100%, a score higher than 40% is considered to indicate an increased fall risk and is used as inclusion criteria for participation in this study. In addition to the overall SI, the hunova robot compares the results of seven dimensions (static balance, dynamic balance, reactive balance, limits of stability, gait speed, sensory integration, and sit to stand) to a reference sample of the same age. Based on this comparison, each dimension is defined as normal (on average) meaning no deficit or having a performance below average resulting in a yellow (poor performance), orange (very poor performance), or red deficit (very very poor performance) for this dimension. The dimensions (and deficits) are visualised in a radar plot (see Fig. 3). These dimensions and deficits are relevant for the planning of the training sessions within the intervention (see ‘intervention’).

#### Mobility test

In addition to the aforementioned tests, the Timed-up-and-go test (TUG) [40] is administered during all planned assessments (T_0_, T_1_, T_2,_ and T_3_) for all included participants.

#### Fall diary

To assess the number of falls, participants are instructed to keep a diary throughout the complete study (i.e., between T_0_ and T_3_). In the context of this study, a fall is defined conform Lamb and colleagues [41] “a fall is an unexpected event in which the participant comes to rest on the ground, floor, or lower level”. In the fall diary, the participant indicates for each day whether one or more of following events [5] occurred; a ‘trip or slip’, a ‘fall without injuries’, or an ‘injurious fall’. In case of a fall, details of the fall are documented in a fall anamnesis questionnaire. After inclusion in the study, participants receive sheets for every month of their study participation. Sheets for completed months are collected every time the participants visit the study centres for assessments.

#### Questionnaires

To evaluate aspects of the intervention, several questionnaires will be administered at T_1_ (after finalising the first part of the intervention) or T_2_ (after finalising the home-based programme), and an exercise diary will be kept:

• The general feedback to the technology-supported exercise programme and the general feedback to the home-based exercise programme are assessed based on custom self-developed questionnaires that aim to assess feasibility;
• The Usefulness, Satisfaction and Ease of Use (USE) questionnaire, a validated and reliable instrument [42], is used to assess user satisfaction with the technology-supported exercise programme and the home-based exercise programme;
• The Task Load Index (TLX), a validated and reliable questionnaire [43], is used to assess subjective effort during the technology-supported exercise programme and the home-based exercise programme;
• The User Experience Questionnaire (UEQ) [44] is used to evaluate both parts of the intervention;
• Based on a customised questionnaire, overall feedback to the study will be collected to assess the subjective evaluation of participating in the study’s intervention.
• An ‘Exercise diary’ is used to evaluate the home-based exercise programme and subjective effort.

Lastly, as additional background information, overall health status and physical activity will be monitored throughout the complete intervention (i.e., between T_0_ and T_3_). Each month, physical activity will be assessed based on the German PAQ-50 + [45], and to assess health status, the EQ-5D-5L [46] will be used at T_0_, T_1_, T_2_ and T_3_.

### Intervention

#### Technology-supported exercise programme (TSEP)

The first intervention period refers to the technology-supported exercise programme (TSEP) using the hunova robot (Movendo Technology, Genoa, Italy). The hunova robot is a programmable robotic medical device that consists of two electromechanical platforms with two degrees of freedom, one at foot level (foot platform) and one at seat level (seat). Both platforms include two motorized axes. The platforms are rigidly connected to the robot movement axes by means of a torque and force sensor (6-axis sensor). The system allows two degrees of freedom of mobility (forwards-backwards = anterior-posterior and left-right = medio-lateral). The hunova robot can operate in static, active, passive, and assistive modes. Further details can be found in additional file 1. The hunova robot operates in conjunction with a wireless sensor (Inertial Movement Unit - IMU) located on the participant’s torso, to monitor torso motion. All exercises are accompanied by graphical and audio applications (providing biofeedback) run by the remote computer, with which the participant interacts to complete the exercises.

The participants are supervised in a 1:1 situation by a sport or physio-therapist for about 30 min per training session. By performing various functional, balance, and strength exercises on the hunova robot, the participants train within their individual deficit areas of balance and muscle strength while receiving visual feedback via a monitor.

The design of the TSEP training is planned and carried out on three levels. The first level is represented by the training areas, the second level is the type of activity and the third is the difficulty of each activity. The TSEP is the result of the combination of the three layers and the detailed description is provided in the following paragraphs. At first, there is the training layer (level 1), based on the seven subsections of the SI supplemented by a maintenance area, which consists of exercises that train the seven functional areas assessed. These eight training areas are named static balance, dynamic balance, sensory integration, reactive balance, gait speed, limits of stability, sit to stand and maintenance. For each dimension, specific exercises are possible with the hunova robot. A detailed description of all exercises implemented within the TSEP using the hunova robot is presented in Table 1.

**Table 1:** Exercise list and descriptions (COP = centre of pressure)

Training area (level 1)	Task description	Position	Mode	Difficulty regulation (level 3)
Static balance	Participant / exercising person must maintain balance while shifting her/his COP through whole body movements	Standing bipodal or standing with one leg on foot platform, one behind	Static foot platform	- load on one leg - hands/torso reaching - inclined plane - dual task exercises - squats - leg raises
Dynamic balance	Participant / exercising person must maintain balance while the foot platform simulates changing terrain and moves in predefined directions/patterns	Standing bipodal or standing with one leg on foot platform, one behind	Active foot platform	- load on one leg - variation in movement planes - higher amplitude of motion - hands reaching - dual task exercises
Sensory integration (dynamic balance + visual integration)	Participant / exercising person must maintain balance on various simulated surfaces using visual-motor or proprioceptive integration	Standing bipodal or sitting	Static, active, passive foot platform, active, passive seat	- eyes closed - reaching - unstable surface - dual task exercises
Reactive balance	Participant / exercising person must maintain balance while the foot platform is movable and follows her/his movements	Standing bipodal or standing with one leg on foot platform, one behind	Active, passive foot platform	- load on one leg - hands/torso reaching - dual task exercises - platform gives impulses
Gait speed (dynamic balance + reactive balance)	Participant / exercising person must maintain balance while the foot platform simulates changing terrain or follows her/his movements	Standing bipodal or standing with one leg on foot platform, one behind	Active, passive foot platform	- load on one leg - platform gives impulses - torso/hands reaching - variation in movement planes
Limits of stability	Participant / exercising person must maintain balance while moving her/his COP through whole body movements following predefined patterns	Standing bipodal	Static, active, passive foot platform	- reaching (with load) - higher amplitude of motion - variation in movement planes - variation in movement patterns
Sit to stand	Participant / exercising person must maintain balance in a sitting position while moving actively or to compensate seat movement	Sitting or standing bipodal	Active, passive seat, static, active foot platform	- increasing seat movement - torso reaching - sit to stand - inclined plane - squats
Maintenance	Participant / exercising person must maintain balance while doing exercises from all training areas	Standing bipodal or standing with one leg on foot platform, one behind or sitting	Static, active, passive foot platform, static, active seat	Regulation through measures from all other training areas

To guarantee training success, basic principles of exercise / training science were considered within the training programme. To progressively increase the load and difficulty, level 2 (activity) and level 3 (difficulty) should be used. Each training area (level 1) is further divided into three different activities, including various exercises. In each activity block, there are ten different exercises resulting in 30 exercises per training area. To progressively increase the requirements alongside the activities, the following parameters are used: number of repetitions and sets, addition of dual task exercises, and reduction of the base of support. On level 3, three different difficulty levels can be used to adjust the load (and difficulty) during the exercises individually according to the number and characteristics of the deficits of the participants / exercising persons. At difficulty level “easy (E)”, each exercise has a movement duration of 30 s, and 3 sets should be performed. In the “medium (M)” difficulty level, each exercise has a movement duration of 45 s, and 2 sets should be performed, ending with “difficult (D)” with 60 s and one set. A total number of exercises of 30 in E, 20 in M, and 15 (7/8 in 2 sets) in D are possible. With this design, a net training time of 15 min should be realised, resulting in a session duration of 30 min per session. In the section of strengthening exercises, the hunova robot automatically gives instructions about movement duration, rest between repetitions and sets. For the sit to stand exercises, the therapists should control for 6 repetitions of the sit to stand exercise during difficulty level E, 8 repetitions for difficulty level M and 2 sets with 6 repetitions for difficulty level D (15 s rest between sets). Within the sit to stand movements, 2 s for concentric, 1 s isometric and 2 s for eccentric movement should be realised.

For the detailed planning and implementation of level 3 (difficulty), the number, the dimensions, and the characteristics (yellow, orange, red) of the deficits in the seven dimensions during the SI measurements must be considered (see additional file 2). In each training session (of the 24 sessions in total), one dimension is focused. Within the total training period, the two worst dimensions (depending on the SI results) are included in the exercise schedule. For standardisation purposes and for better comparability between persons, there is a fixed training progression. For this purpose, a manual was created for the sport and physio-therapists that contains every potential case that can occur within the SI measurements. It also covers the next steps for training planning and implementation. All details about this design are presented in additional file 3. If the planned training in the TSEP is too difficult for a participant, the (starting) difficulty level can be lowered by the therapists, the progression of training difficulty remains the same for these participants.

#### Home-based exercise programme (HBEP)

The second intervention block implements a home-based training programme (HBEP). The training with various balance, functional, and strength exercises aimed to prevent falls by furthering the training progress made in the first intervention block using the TSEP. All home exercises are based on the exercises in the TSEP but do not require any technological equipment. With functional exercises such as heel and toe walk, squats, knee raises, calf raises, tandem walk and lunges, aspects of gait and lower limb strength relevant to everyday life will be trained. The difficulty of the exercises is progressively increased over the course of the intervention period via the following parameters: number of repetitions and sets, inclusion of dual task exercises, and reduction of the base of support. All exercises and training details are summarised in a manual. The instructions of the exercises for the 24 training sessions are given, on the one hand, via this manual and, on the other hand, via provided videos. The videos are provided online through a website. Based on the participants’ preferences and technical prerequisites, they can use their preferred means of training. In addition, the first two training sessions of HBEP take place in the therapy centres with one-to-one supervision (= 26 supervised training sessions in the study centres).

Within the HBEP period for participants of the INT group and between T_2_ and T_3_ for participants of both groups (INT and CTR), phone calls are made regularly for motivation, potential questions, and feedback. For additional motivation to participate in the study until the last measurement (T3), participants in both groups will receive vouchers for ongoing study participation (20€ after T1 measurement, 30€ after T2 measurement, and 50€ after T3 measurement).

### Statistical analyses

#### A-priori estimation of sample size

Based on Sherrington and colleagues [10], it is assumed that the intervention will reduce the number of falls by 23% in comparison to the control group. Based on recommendations by Robertson [47], a binominal regression analysis was used to estimate the required sample size. Using G*Power [48, 49], a regression analysis with group (INT/CTR) as independent variable and number of falls as dependent variable, and -23% as the assumed reduction of falls, yielded a required total sample size of 181 persons. Assuming that the study will have an overall dropout rate of 30% [10], a total number of 235 participants to be included in the study is estimated.

#### Planned statistical evaluation of outcomes

An analysis of primary outcomes (i.e., number of falls and number of persons who have fallen) will be based on binomial regression analyses with demographic variables, physical activity, and health parameters as covariates. Analyses of secondary outcomes (SI and TUG) will be based on 2 × 4 ANOVA / ANCOVA with groups (INT/CTR) and time of assessment (T_0_, T_1_, T_2_ and T_3_) as factors, and will consider demographic variables, physical activity, and health parameters as co-variates. Significant main or interaction effects will be investigated further using Bonferroni post-hoc-tests. The significance level is set to *p* < 0.05.

## Discussion

### Expected key results

Important predictors of fall risk, such as muscle function, balance control and gait quality, can be improved by appropriate fall prevention interventions [10, 11]. Effective fall prevention strategies should simultaneously address different risk factors [10, 12, 13]. Exercise-based strategies are most effective when they combine balance exercises and functional exercises optionally supplemented with strength training [10].

Although several studies in recent years used miscellaneous new technology within novel fall prevention interventions [23–28], so far, the potential contribution of the hunova robot (Movendo Technology, Genoa, Italy) to reducing fall risk has not been investigated. Based on clinical studies that demonstrated positive results in the rehabilitation of stroke patients as well as in patients with Parkinson’s disease (PD) [37, 38]. Cella et al. [35] demonstrated that the hunova robot can discriminate and evaluate older patients and predict falls. Furthermore, the hunova assessment can discriminate older persons with different grades of impairment of physical performance [50]. With this in mind, the hunova robot seems to be suitable for fall prevention. With its specific assessment strategy which results in an individual fall risk score (SI) and the possibility to create an individually tailored specific exercise programme based on the deficits in the dimensions, the hunova robot provides a new approach to fall prevention. To ensure training success, basic principles of exercise / training science were considered within the training programme.

Based on this planning, we expect positive changes regarding the overall SI but also in the seven subsections (dimensions) addressing different risk factors for falls and for the timed up-and-go test. First positive results on risk factors can be expected after completing the first 24 training sessions using the hunova robot. After this period, an additional 24 training sessions lasting period of home-based training should sustain or even expand the positive changes in the following time. With 48 training sessions over at least 6 months, fall risk factors should positively be influenced in the participants. However, as primary outcomes, the number of falls and fallers within the training period and in a one-year follow-up period are the most relevant parameters. We believe that by applying our training programme, older adults having an increased fall risk (SI > 40%) can experience relevant improvements in functional performance, tested within different dimensions of fall risk, and a reduced number of falls and fallers as primary outcomes, compared to the control group.

### Benefits and risks

Participants who take part in the fall prevention training programme using the hunova robot and the subsequent home-based training programme have potential benefits from an individually tailored training programme that takes basic principles of exercise / training science into account. No adverse events are expected during the measurements or the intervention using the hunova robot and the first two training sessions of the home-based programme because of the supervision of trained sport or physio-therapists who are trained with all details of the training programme in advance. In addition, all training content is developed based on current knowledge of training science, e.g., having a progression over time, first balance exercises and strengthening exercises at the end. During the supervised training sessions, participants are always made aware of potential hazards and learn how to assist themselves, e.g., during balance exercises, or having problems within an exercise. In addition, different potential adjustments, and modifications to simplify and to complicate the exercises are given by the therapists. With this preparation over the first 26 training sessions (supervised by the therapists in the study centres: 24 sessions TSEP + 2 sessions HBEP = 26 sessions on-site), having the manual and the accompanying videos, participants should be optimally prepared for the home-based training programme in the second period. By implementing the health questionnaire and carefully selected inclusion and exclusion criteria, all participants are expected to fulfil the prerequisites for study participation. Measurements and training sessions are instructed and supervised by well-trained personnel, so that potential risks are minimised. Proper safety precautions additionally minimize the risk of uncomfortable and adverse events. In summary, the expected benefits exceed the potentially occurring risks.

### Potential limitations and risk for bias

The recruitment of potential participants will be via Generali Health Solutions (GHS), who will address insured older persons of “Generali Deutschland Krankenversicherung AG” (Generali Germany Health Insurance AG) and its subsidiary who live in the close area of the participating study centres. They receive detailed study information. The recruitment process could potentially lead to a selective recruitment of those older adults who are intrinsically motivated to exercise and who are already physically active. The fact that our study included older adults with a higher fall risk (according to the Silver Index, > 40%), while still being able to do 30 min of physical activity, could potentially bias our results. This aspect should be kept in mind while interpreting the results of the study. All interested persons had to state that they were willing to be randomised in one of the two groups to minimise dropouts, particularly in the control group. Within the randomisation procedure, a random allocation of all available places for the study is made by drawing lots. The examiners of the centres have no influence on the allocation to the groups, a change between groups is not possible and group preferences are strictly not allowed. Since the therapists do both measurements and the supervision of the training sessions, blinding of assessors is not possible which yields an acceptable risk for bias. The measurements at T_0_, T_1_, T_2_ and T_3_ are standardised and performed in the same room in each study centre by the same assessor. Measurements should be scheduled at the same day of the week and at the same time of the day for the four measurements if possible. The control group does not receive any training treatment, but they obtain the opportunity to use an optimised training programme after the study is finished (including 1-year follow-up). The participants in the control group are instructed to maintain their usual physical activity during the study. Due to the potential bias and influence of different levels of physical activity, the amount is documented through the German PAQ-50 + during the study duration for both groups. With having a multi-centre study, a bias of regional differences should be minimised. Furthermore, health status of older adults can change over the study duration and influence participation, drop-out rate and results of the study. To gain insights here, the EQ-5D-5L is implemented.

This study is expected to lead to new insights which may help establish a new approach to fall prevention training for older adults at risk for falls. Based on the results of the first part of the intervention, adjustments of load criteria, training content, and exercises can be realised for further using of this approach. Together with the SI measurement, the approach includes a systematic and individually tailored innovative fall prevention programme that can be implemented afterwards in a broad fall-prone target group of older adults. The results will be published in a peer-reviewed scientific journal to make them accessible to the scientific community. After the study completion, approaches to examine the cost-effectiveness and develop an implementation plan are relevant aspects for further steps.

## Supplementary Information

**Additional file 1.** Terms and possibilities of the hunova robot device for variations in the exercise schedule.

**Additional file 2.** Details of the setting for planning and implementing the training sessions using the hunova robot regarding number of deficits, number of suggested training dimensions, dimensions, number of exercises, and duration of the training session.

**Additional file 3.** Number of deficits and their colours during the Silver Index (SI) Measurement and information about how to go on for exercise planning using these characteristics (C = case).

## Data Availability

Data sharing is not applicable yet to this article as participant recruitment is still ongoing. The datasets generated and/or analysed during the implementation of the study protocol are available from the corresponding author on reasonable request.

## Abbreviations

CTR: Control group
COP: Centre of pressure
COPD: Chronic obstructive pulmonary disease
D: Difficult
E: Easy
FTSST: Five times sit to stand test
GHS: Generali Health Solutions
GSU: German Sport University Cologne
HBEP: Home-based exercise programme
IMU: Inertial movement unit
INT: Intervention group
M: Medium
PAQ: Physical activity questionnaire
PD: Parkinson’s disease
RCT: Randomised controlled trial
SI: Silver Index
TLX: The task load index questionnaire
TSEP: Technology-supported exercise programme
TUG: Timed-up-and-go test
UEQ: User experience questionnaire
USE: Usefulness, satisfaction and ease of use questionnaire
