# Supplementary material for: Evaluation of a novel technology-supported fall prevention intervention – study protocol of a multi-centre randomised controlled trial in older adults at increased risk of falls
Source: BMC Geriatr. 2023 Feb 18;23:103. doi: 10.1186/s12877-023-03810-8 (PMC9938567; doi:10.1186/s12877-023-03810-8)
Supplement: Supplementary file 1 — Additional file 1. Terms and possibilities of the hunova robot device for variations in the exercise schedule. [file 12877_2023_3810_MOESM1_ESM.docx]

**Additional file 1.** Terms and possibilities of the hunova robot device for variations in the exercise schedule.

The hunova robot enables various functions in four different groups, all details can be found in the hunova user manual.

1. Static: The foot platform / seat remains stationary and simulates a static / flat surface. Within the static mode, an inclined plane is one extra feature: The foot platform / seat tilts in a given / defined direction and with a certain amplitude to simulate an inclined plane.
2. Active: The participant / exercising person actively moves the (passive) robotic platforms. The foot platform / seat follows the movement of the participant / exercising person and can oppose the movement by providing an adjustable resistance [in %]. Using the active mode, several specific sub settings are possible:
   1. Proprioceptive or wobbling: The foot platform / seat is free and simulates the behaviour of a traditional balance board. In addition, one plane of movement can be locked to obtain pivots on a single axis (e.g. frontal, sagittal plane).
   2. Elastic: The foot platform / seat generates a resistance that simulates exercising with elastic bands.
   3. Fluid: The foot platform / seat generates a resistance that simulates a movement through fluid.
   4. Counter-resistance: Within this mode, the participant / exercising person must exercise against a constant resistance which is generated by the foot platform / seat in a defined direction
3. Passive: The hunova robot imposes a movement and passively moves the participant / exercising person. Using the passive mode, the following sub settings are possible:
   1. Continuous passive mobilisation: The foot platform / seat evokes a pre-defined movement to the participant / exercising person. This is possible in a rectilinear trajectory on a single plane or in a combined trajectory (circle, vertical or horizontal ellipse, infinite trajectories, or figures of 8, spiral).
   2. Impulsive perturbation: The foot platform / seat induces random impulsive movements in the frontal or in the sagittal plane, or in diagonal directions within a pre-defined range of motion [in ° = degrees]. The participant / exercising person has the task to react on the perturbations, to stabilize themselves and maintain their balance.
4. Assistive: The hunova robot helps the participant / exercising person to complete the exercises when its sensors detect problems / struggling within solving the task of the exercise.
