# Supplementary material for: Evaluation of a novel technology-supported fall prevention intervention – study protocol of a multi-centre randomised controlled trial in older adults at increased risk of falls
Source: BMC Geriatr. 2023 Feb 18;23:103. doi: 10.1186/s12877-023-03810-8 (PMC9938567; doi:10.1186/s12877-023-03810-8)
Supplement: Supplementary file 2 — Additional file 2. Details of the setting for planning and implementing the training sessions using the hunova robot regarding number of deficits, number of suggested training dimensions, dimensions, number of exercises, and duration of the training session. [file 12877_2023_3810_MOESM2_ESM.docx]

**Additional file 2.** Details of the setting for planning and implementing the training sessions using the hunova robot regarding number of deficits, number of suggested training dimensions, dimensions, number of exercises, and duration of the training session.

| number of deficit areas | number of suggested training dimensions | dimensions | number of exercises | duration of the training session |
| --- | --- | --- | --- | --- |
|  |  |  |  |  |
|  |  |  |  |  |
| 0 | 1 | fall risk - maintenance | 17 | 35 min + ankle configuration changes |
| 1 | 1 | fall risk | 8-12 | 16-24 min |
|  | 2 (if deficit area is gait or sensory integration) | fall risk - dynamic balance fall risk - reactive balance OR sensory integration | 19-23 (minus duplicate exercises) | 38-46 min |
| 2 | 2 | fall risk fall risk | 8-12 per dimension | 16-24 min per dimension |
|  | 3 (if at least 1 deficit area is gait or sensory integration) | fall risk fall risk - dynamic balance fall risk - reactive balance OR sensory integration | 8-12 (for deficit areas that are not gait or sensory integration)  19-23 (minus duplicate exercises) (for gait / sensory integration) | 16-24 min (for deficit areas that are not gait / sensory integration) 38-46 min (for gait / sensory integration) |
|  | 4 (if gait and sensory integration are the deficit areas) | fall risk - dynamic balance fall risk - reactive balance fall risk - dynamic balance fall risk - sensory integration | 19-23 per session (minus duplicate exercises) | 38-46 min per session |
| 3 or more: select the 2 worst areas from the radar plot and do what is described in the case above (2 deficit areas) | | | | |
