# Supplementary material for: Evaluation of a novel technology-supported fall prevention intervention – study protocol of a multi-centre randomised controlled trial in older adults at increased risk of falls
Source: BMC Geriatr. 2023 Feb 18;23:103. doi: 10.1186/s12877-023-03810-8 (PMC9938567; doi:10.1186/s12877-023-03810-8)
Supplement: Supplementary file 3 — Additional file 3. Number of deficits and their colours during the Silver Index (SI) Measurement and information about how to go on for exercise planning using these characteristics (C = case). [file 12877_2023_3810_MOESM3_ESM.docx]

**Additional file 3.** Number of deficits and their colours during the Silver Index (SI) Measurement and information about how to go on for exercise planning using these characteristics (C = case).

No deficit

|  | 1 | 2 | 3 | 4 | 5 | 6 | 7 | table |
| --- | --- | --- | --- | --- | --- | --- | --- | --- |
| C1 |  |  |  |  |  |  |  | L |

1 deficit

|  | 1 | 2 | 3 | 4 | 5 | 6 | 7 | table |
| --- | --- | --- | --- | --- | --- | --- | --- | --- |
| C1 |  |  |  |  |  |  |  | A |
| C2 |  |  |  |  |  |  |  | B |
| C3 |  |  |  |  |  |  |  | C |

2 deficits

|  | 1 | 2 | 3 | 4 | 5 | 6 | 7 | table |
| --- | --- | --- | --- | --- | --- | --- | --- | --- |
| C1 |  |  |  |  |  |  |  | D |
| C2 |  |  |  |  |  |  |  | E |
| C3 |  |  |  |  |  |  |  | F |
| C4 |  |  |  |  |  |  |  | G |
| C5 |  |  |  |  |  |  |  | H |
| C6 |  |  |  |  |  |  |  | I |

3 deficits

|  | 1 | 2 | 3 | 4 | 5 | 6 | 7 | table | additional steps |
| --- | --- | --- | --- | --- | --- | --- | --- | --- | --- |
| C1 |  |  |  |  |  |  |  | D | Use the two worst yellow deficits and follow table D |
| C2 |  |  |  |  |  |  |  | E | Use the two worst orange deficits and follow table E |
| C3 |  |  |  |  |  |  |  | J | Use the three red deficits and follow table J |
| C4 |  |  |  |  |  |  |  | F | Use the red deficits and follow table F |
| C5 |  |  |  |  |  |  |  | F | Use the red deficits and follow table F |
| C6 |  |  |  |  |  |  |  | G | Use the red deficit and the worst orange deficit and follow table G |
| C7 |  |  |  |  |  |  |  | G | Use the red deficit and the orange deficit and follow table G |
| C8 |  |  |  |  |  |  |  | H | Use the red deficit and the worst yellow deficit and follow table H |
| C9 |  |  |  |  |  |  |  | E | Use the orange deficits and follow table E |
| C10 |  |  |  |  |  |  |  | I | Use the orange deficit and the worst yellow deficit and follow table I |

4 deficits

|  | 1 | 2 | 3 | 4 | 5 | 6 | 7 | table | additional steps |
| --- | --- | --- | --- | --- | --- | --- | --- | --- | --- |
| C1 |  |  |  |  |  |  |  | D | Use the two worst yellow deficits and follow table D |
| C2 |  |  |  |  |  |  |  | E | Use the two worst orange deficits and follow table E |
| C3 |  |  |  |  |  |  |  | K | Use the four red deficits and follow table K |
| C4 |  |  |  |  |  |  |  | J | Use the three red deficits and follow table J |
| C5 |  |  |  |  |  |  |  | J | Use the three red deficits and follow table J |
| C6 |  |  |  |  |  |  |  | F | Use the two red deficits and follow table F |
| C7 |  |  |  |  |  |  |  | F | Use the two red deficits and follow table F |
| C8 |  |  |  |  |  |  |  | F | Use the two red deficits and follow table F |
| C9 |  |  |  |  |  |  |  | G | Use the red deficit and the worst orange deficit and follow table G |
| C10 |  |  |  |  |  |  |  | H | Use the red deficit and the worst yellow deficit and follow table H |
| C11 |  |  |  |  |  |  |  | G | Use the red deficit and the worst orange deficit and follow table G |
| C12 |  |  |  |  |  |  |  | G | Use the red and orange deficit and follow table G |
| C13 |  |  |  |  |  |  |  | E | Use the two worst orange deficits and follow table E |
| C14 |  |  |  |  |  |  |  | E | Use the orange deficits and follow table E |
| C15 |  |  |  |  |  |  |  | I | Use the orange deficit and the worst yellow deficit and follow table I |

more than 4 deficits

|  | 1 | 2 | 3 | 4 | 5 | 6 | 7 | additional steps |
| --- | --- | --- | --- | --- | --- | --- | --- | --- |
| C X |  |  |  |  |  |  |  | Identify the 4 worst deficits and identify your case in the table "4 deficits" and follow the additional steps |

# Table A (1 yellow deficit)

yellow deficit: _________________________________________________

| **session** | **deficit** | **activity** | **difficulty level** |
| --- | --- | --- | --- |
| 1 | yellow | 2 | M |
| 2 | maintenance | 1 | M |
| 3 | yellow | 2 | M |
| 4 | maintenance | 1 | M |
| 5 | yellow | 2 | D |
| 6 | maintenance | 1 | D |
| 7 | yellow | 2 | D |
| 8 | maintenance | 1 | D |
| 9 | yellow | 2 | D |
| 10 | maintenance | 2 | M |
| 11 | yellow | 2 | D |
| 12 | maintenance | 2 | M |
| 13 | yellow | 3 | M |
| 14 | maintenance | 2 | D |
| 15 | yellow | 3 | M |
| 16 | maintenance | 2 | D |
| 17 | yellow | 3 | D |
| 18 | maintenance | 3 | M |
| 19 | yellow | 3 | D |
| 20 | maintenance | 3 | M |
| 21 | yellow | 3 | D |
| 22 | maintenance | 3 | D |
| 23 | yellow | 3 | D |
| 24 | maintenance | 3 | D |

**Table B (1 orange deficit)**

orange deficit: ________________________

| **session** | **deficit** | **activity** | **difficulty level** |
| --- | --- | --- | --- |
| 1 | orange | 1 | M |
| 2 | maintenance | 1 | M |
| 3 | orange | 1 | M |
| 4 | maintenance | 1 | M |
| 5 | orange | 1 | D |
| 6 | maintenance | 1 | D |
| 7 | orange | 1 | D |
| 8 | maintenance | 1 | D |
| 9 | orange | 2 | M |
| 10 | maintenance | 2 | M |
| 11 | orange | 2 | M |
| 12 | maintenance | 2 | M |
| 13 | orange | 2 | D |
| 14 | maintenance | 2 | D |
| 15 | orange | 2 | D |
| 16 | maintenance | 2 | D |
| 17 | orange | 3 | M |
| 18 | maintenance | 3 | M |
| 19 | orange | 3 | M |
| 20 | maintenance | 3 | M |
| 21 | orange | 3 | D |
| 22 | maintenance | 3 | D |
| 23 | orange | 3 | D |
| 24 | maintenance | 3 | D |

# Table C (1 red deficit)

red deficit: _________________

| **session** | **deficit** | **activity** | **difficulty level** |
| --- | --- | --- | --- |
| 1 | red | 1 | E |
| 2 | maintenance | 1 | M |
| 3 | red | 1 | E |
| 4 | maintenance | 1 | M |
| 5 | red | 1 | M |
| 6 | maintenance | 1 | D |
| 7 | red | 1 | M |
| 8 | maintenance | 1 | D |
| 9 | red | 1 | D |
| 10 | maintenance | 2 | M |
| 11 | red | 1 | D |
| 12 | maintenance | 2 | M |
| 13 | red | 2 | E |
| 14 | maintenance | 2 | D |
| 15 | red | 2 | E |
| 16 | maintenance | 2 | D |
| 17 | red | 2 | M |
| 18 | maintenance | 3 | M |
| 19 | red | 2 | M |
| 20 | maintenance | 3 | M |
| 21 | red | 2 | M |
| 22 | maintenance | 3 | D |
| 23 | red | 2 | M |
| 24 | maintenance | 3 | D |

# Table D (2 yellow deficits)

1. yellow deficit: _________________________________

2. yellow deficit: _________________________________

| **session** | **deficit** | **activity** | **difficulty level** |
| --- | --- | --- | --- |
| 1 | yellow1 | 2 | M |
| 2 | yellow2 | 2 | M |
| 3 | yellow1 | 2 | M |
| 4 | yellow2 | 2 | M |
| 5 | yellow1 | 2 | D |
| 6 | yellow2 | 2 | D |
| 7 | yellow1 | 2 | D |
| 8 | yellow2 | 2 | D |
| 9 | yellow1 | 2 | D |
| 10 | yellow2 | 2 | D |
| 11 | yellow1 | 2 | D |
| 12 | yellow2 | 2 | D |
| 13 | yellow1 | 3 | M |
| 14 | yellow2 | 3 | M |
| 15 | yellow1 | 3 | M |
| 16 | yellow2 | 3 | M |
| 17 | yellow1 | 3 | D |
| 18 | yellow2 | 3 | D |
| 19 | yellow1 | 3 | D |
| 20 | yellow2 | 3 | D |
| 21 | yellow1 | 3 | D |
| 22 | yellow2 | 3 | D |
| 23 | yellow1 | 3 | D |
| 24 | yellow2 | 3 | D |

# Table E (2 orange deficits)

1. orange deficit: _________________________________

2. orange deficit: _________________________________

| **session** | **deficit** | **activity** | **difficulty level** |
| --- | --- | --- | --- |
| 1 | orange1 | 1 | M |
| 2 | orange2 | 1 | M |
| 3 | orange1 | 1 | M |
| 4 | orange2 | 1 | M |
| 5 | orange1 | 1 | D |
| 6 | orange2 | 1 | D |
| 7 | orange1 | 1 | D |
| 8 | orange2 | 1 | D |
| 9 | orange1 | 2 | M |
| 10 | orange2 | 2 | M |
| 11 | orange1 | 2 | M |
| 12 | orange2 | 2 | M |
| 13 | orange1 | 2 | D |
| 14 | orange2 | 2 | D |
| 15 | orange1 | 2 | D |
| 16 | orange2 | 2 | D |
| 17 | orange1 | 3 | M |
| 18 | orange2 | 3 | M |
| 19 | orange1 | 3 | M |
| 20 | orange2 | 3 | M |
| 21 | orange1 | 3 | D |
| 22 | orange2 | 3 | D |
| 23 | orange1 | 3 | D |
| 24 | orange2 | 3 | D |

# Table F (2 red deficits)

1. red deficit: _________________________________

2. red deficit: _________________________________

| **session** | **deficit** | **activity** | **difficulty level** |
| --- | --- | --- | --- |
| 1 | red1 | 1 | E |
| 2 | red2 | 1 | E |
| 3 | red1 | 1 | E |
| 4 | red2 | 1 | E |
| 5 | red1 | 1 | M |
| 6 | red2 | 1 | M |
| 7 | red1 | 1 | M |
| 8 | red2 | 1 | M |
| 9 | red1 | 1 | D |
| 10 | red2 | 1 | D |
| 11 | red1 | 1 | D |
| 12 | red2 | 1 | D |
| 13 | red1 | 2 | E |
| 14 | red2 | 2 | E |
| 15 | red1 | 2 | E |
| 16 | red2 | 2 | E |
| 17 | red1 | 2 | M |
| 18 | red2 | 2 | M |
| 19 | red1 | 2 | M |
| 20 | red2 | 2 | M |
| 21 | red1 | 2 | D |
| 22 | red2 | 2 | D |
| 23 | red1 | 2 | D |
| 24 | red2 | 2 | D |

# Table G (2 deficits: red and orange)

red deficit: _________________________________

orange deficit: _________________________________

| **session** | **deficit** | **activity** | **difficulty level** |
| --- | --- | --- | --- |
| 1 | red | 1 | E |
| 2 | orange | 1 | M |
| 3 | red | 1 | E |
| 4 | orange | 1 | M |
| 5 | red | 1 | M |
| 6 | orange | 1 | D |
| 7 | red | 1 | M |
| 8 | orange | 1 | D |
| 9 | red | 1 | D |
| 10 | orange | 2 | M |
| 11 | red | 1 | D |
| 12 | orange | 2 | M |
| 13 | red | 2 | E |
| 14 | orange | 2 | D |
| 15 | red | 2 | E |
| 16 | orange | 2 | D |
| 17 | red | 2 | M |
| 18 | orange | 3 | M |
| 19 | red | 2 | M |
| 20 | orange | 3 | M |
| 21 | red | 2 | D |
| 22 | orange | 3 | D |
| 23 | red | 2 | D |
| 24 | orange | 3 | D |

# Table H (2 deficits: red and yellow)

red deficit: _________________________________

yellow deficit: _________________________________

| **session** | **deficit** | **activity** | **difficulty level** |
| --- | --- | --- | --- |
| 1 | red | 1 | E |
| 2 | yellow | 2 | M |
| 3 | red | 1 | E |
| 4 | yellow | 2 | M |
| 5 | red | 1 | M |
| 6 | yellow | 2 | D |
| 7 | red | 1 | M |
| 8 | yellow | 2 | D |
| 9 | red | 1 | D |
| 10 | yellow | 2 | D |
| 11 | red | 1 | D |
| 12 | yellow | 2 | D |
| 13 | red | 2 | E |
| 14 | yellow | 3 | M |
| 15 | red | 2 | E |
| 16 | yellow | 3 | M |
| 17 | red | 2 | M |
| 18 | yellow | 3 | D |
| 19 | red | 2 | M |
| 20 | yellow | 3 | D |
| 21 | red | 2 | D |
| 22 | yellow | 3 | D |
| 23 | red | 2 | D |
| 24 | yellow | 3 | D |

# Table I (2 deficits: orange and yellow)

red deficit: _________________________________

yellow deficit: _________________________________

| **session** | **deficit** | **activity** | **difficulty level** |
| --- | --- | --- | --- |
| 1 | orange | 1 | M |
| 2 | yellow | 2 | M |
| 3 | orange | 1 | M |
| 4 | yellow | 2 | M |
| 5 | orange | 1 | D |
| 6 | yellow | 2 | D |
| 7 | orange | 1 | D |
| 8 | yellow | 2 | D |
| 9 | orange | 2 | M |
| 10 | yellow | 2 | D |
| 11 | orange | 2 | M |
| 12 | yellow | 2 | D |
| 13 | orange | 2 | D |
| 14 | yellow | 3 | M |
| 15 | orange | 2 | D |
| 16 | yellow | 3 | M |
| 17 | orange | 3 | M |
| 18 | yellow | 3 | D |
| 19 | orange | 3 | M |
| 20 | yellow | 3 | D |
| 21 | orange | 3 | D |
| 22 | yellow | 3 | D |
| 23 | orange | 3 | D |
| 24 | yellow | 3 | D |

# Table J (3 red deficits)

1. red deficit: _________________________________

2. red deficit: _________________________________

3. red deficit: _________________________________

| **session** | **deficit** | **activity** | **difficulty level** |
| --- | --- | --- | --- |
| 1 | red1 | 1 | E |
| 2 | red2 | 1 | M |
| 3 | red3 | 1 | E |
| 4 | red1 | 1 | E |
| 5 | red2 | 1 | E |
| 6 | red3 | 1 | E |
| 7 | red1 | 1 | M |
| 8 | red2 | 1 | M |
| 9 | red3 | 1 | M |
| 10 | red1 | 1 | M |
| 11 | red2 | 1 | M |
| 12 | red3 | 1 | M |
| 13 | red1 | 2 | E |
| 14 | red2 | 2 | E |
| 15 | red3 | 2 | E |
| 16 | red1 | 2 | E |
| 17 | red2 | 2 | E |
| 18 | red3 | 2 | E |
| 19 | red1 | 2 | M |
| 20 | red2 | 2 | M |
| 21 | red3 | 2 | M |
| 22 | red1 | 2 | M |
| 23 | red2 | 2 | M |
| 24 | red3 | 2 | M |

# Table K (4 red deficits)

1. red deficit: _________________________________

2. red deficit: _________________________________

3. red deficit: _________________________________

4. red deficit: _________________________________

| **session** | **deficit** | **activity** | **difficulty level** |
| --- | --- | --- | --- |
| 1 | red1 | 1 | E |
| 2 | red2 | 1 | M |
| 3 | red3 | 1 | E |
| 4 | red4 | 1 | E |
| 5 | red1 | 1 | E |
| 6 | red2 | 1 | E |
| 7 | red3 | 1 | E |
| 8 | red4 | 1 | E |
| 9 | red1 | 1 | M |
| 10 | red2 | 1 | M |
| 11 | red3 | 1 | M |
| 12 | red4 | 1 | M |
| 13 | red1 | 1 | M |
| 14 | red2 | 1 | M |
| 15 | red3 | 1 | M |
| 16 | red4 | 1 | M |
| 17 | red1 | 1 | M |
| 18 | red2 | 2 | E |
| 19 | red3 | 2 | E |
| 20 | red4 | 2 | E |
| 21 | red1 | 2 | E |
| 22 | red2 | 2 | E |
| 23 | red3 | 2 | E |
| 24 | red4 | 2 | E |

#

# Table L (no deficit)

| **session** | **deficit** | **activity** | **difficulty level** |
| --- | --- | --- | --- |
| 1 | maintenance | 1 | M |
| 2 | maintenance | 1 | M |
| 3 | maintenance | 1 | M |
| 4 | maintenance | 1 | M |
| 5 | maintenance | 1 | D |
| 6 | maintenance | 1 | D |
| 7 | maintenance | 1 | D |
| 8 | maintenance | 1 | D |
| 9 | maintenance | 2 | M |
| 10 | maintenance | 2 | M |
| 11 | maintenance | 2 | M |
| 12 | maintenance | 2 | M |
| 13 | maintenance | 2 | D |
| 14 | maintenance | 2 | D |
| 15 | maintenance | 2 | D |
| 16 | maintenance | 2 | D |
| 17 | maintenance | 3 | M |
| 18 | maintenance | 3 | M |
| 19 | maintenance | 3 | M |
| 20 | maintenance | 3 | M |
| 21 | maintenance | 3 | D |
| 22 | maintenance | 3 | D |
| 23 | maintenance | 3 | D |
| 24 | maintenance | 3 | D |

# 
